# Oxidation of Small Phenolic Compounds by Mn(IV)

**DOI:** 10.3390/molecules29184320

**Published:** 2024-09-12

**Authors:** Madeline G. Gruenberg, Jonathan J. Halvorson, Ann E. Hagerman, Ikponmwosa G. Enoma, Michael A. Schmidt

**Affiliations:** 1 Department of Biochemistry and Molecular Biology, Wright State University, Dayton, OH 45435, USA; gruenberg.3@wright.edu (M.G.G.);; 2 Northern Great Plains Research Laboratory, United States Department of Agriculture—Agricultural Research Service, Mandan, ND 58554, USA; jonathan.halvorson@usda.gov; 3 Department of Chemistry & Biochemistry, Miami University, Oxford, OH 45056, USA; hagermae@miamioh.edu

**Keywords:** phenolic acids, oxidation, manganese, soil organic matter, NMR, HPLC, CO_2_

## Abstract

Plant secondary metabolites, including phenolics, represent a large quantity of organic material that enters soil and contributes to the formation of soil organic matter (SOM). The process of phenolics forming SOM remains poorly understood. One possible mechanism is oxidation of the phenolic compound catalyzed by redox active metals such as manganese (Mn) and iron (Fe) in soils. In this work, we report how three phenolic compounds react with a redox active environmentally relevant metal, Mn(IV). The reactions were monitored via nuclear magnetic resonance (NMR), high-performance liquid chromatography (HPLC), and direct CO_2_ measurements. Using these techniques, we demonstrate that gallic acid reacts with Mn(IV) less efficiently than pyrogallol. The products of the gallic acid:Mn(IV) reaction are more oxidized than the products of the pyrogallol reaction. Gallic acid produces small molecules and releases CO_2_, while pyrogallol produces a less oxidized product, likely a quinone, and releases less CO_2_. Benzoic acid did not react with Mn(IV). This work provides a framework for how different classes of plant secondary metabolites may be degraded abiotically by redox active metals in soil.

## 1. Introduction

Plant secondary metabolites are a diverse class of compounds that range from small organic molecules to large polymers [1,2]. The exact mixture of secondary metabolites produced by a plant is dependent on the genetics, stress, and the environment [3,4]. These compounds enter the soil through several mechanisms such as root exudates, rain runoff, or decomposition products [5,6]. Once in the soil, they play a major role in several processes, including formation of soil organic matter and reacting with soil metals [7,8,9,10].

Manganese (Mn) is an essential mineral for plant health and plays a critical role in photosynthesis [11]. Mn in soil exists in several states, including the soluble Mn^2+^ and insoluble Mn oxides. Due to the their oxidation potential and surface area, Mn oxides play a significant role in soil redox reactions contributing to soil organic matter and litter decomposition [12]. Both biotic degradation via Mn-dependent enzymes and abiotic oxidation of organic compounds by reactive Mn in soil can contribute to SOM [12]. Several reports have demonstrated that the rate of litter decomposition, as measured by mass loss, is dependent on the concentration of Mn, with more litter decomposition in samples with higher reactive Mn oxide concentrations [13,14]. The amount of litter carbon that is made into SOM rather than CO_2_ is likely dependent on several factors, such as microbial respiration, temperature, moisture, and plant secondary metabolite inputs [15].

One important group of plant secondary metabolites is phenolics, which include smaller compounds such as phenolic acids and polymeric polyphenolics such as tannins and lignins. Phenolic acids are produced by many different types of plants. They are classified into two major types, benzoic acid or cinnamic acid derivatives. The benzoic acid derivatives are aromatic carboxylic acids, while the cinnamic acid derivates have an unsaturated carboxylic acid side chain [16]. The release of phenolic acids from plants and the amount remaining in the soil has been examined [17]. The exact amount of phenolic acids released is dependent on the plant; however, a recent study found over 315 μg of phenolic acids per gram of plant litter, which decreased substantially during the initial 3 months of decomposition [17]. Although the loss of the phenolics is well documented, the process of degradation has not been widely investigated. Gallic acid, 3,4,5-trihydroxybenzoic acid, has been studied in both health and environmental processes due to its widespread distribution in plants and its antioxidant activity [18,19]. As a result of gallic acid being broadly investigated, we selected it to be the model compound for our studies of how phenolics are degraded by Mn(IV).

**Figure 1:**
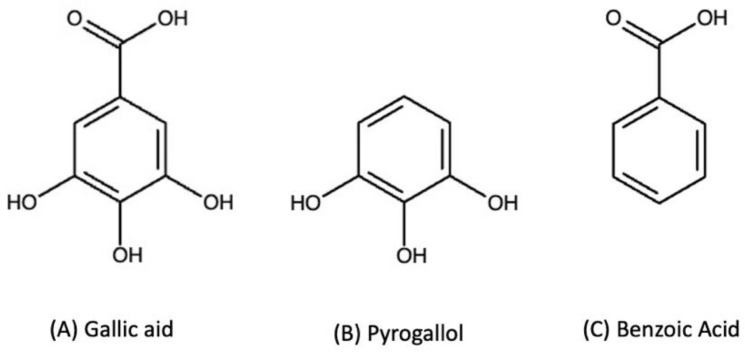
Chemical structures of the phenol compounds.

Recently, we observed that application of gallic acid to soil elicited an abiotic production of CO_2_ [20]. In this same report, we determined that gallic acid produced CO_2_ in the presence of metal oxides, with Mn(IV) producing the most CO_2_ from the gallic acid treatment when compared to the other metal oxides examined. Our subsequent hypothesis was that the CO_2_ released during this reaction was from a simple decarboxylation of the carboxylic acid group, leaving the ring structure intact and forming either pyrogallol or a quinone. Here, we test that hypothesis using two additional model compounds, pyrogallol and benzoic acid. Pyrogallol is a benzene ring with hydroxy groups at positions 1, 2, and 3 (Figure 1). This structure is similar to gallic acid, but is missing the carboxylic acid group. Benzoic acid has the carboxylic acid functionality, but does not have the hydroxyl groups of gallic acid (Figure 1). By using these three compounds, we can systematically assess the role of the carboxylic acid and hydroxyl functionalities in the reaction. We reacted these three compounds with Mn(IV), the strongest producer of CO_2_ in our previous study, and monitored the reactions using NMR spectroscopy, HPLC, and direct CO_2_ measurements.

## 2. Results

### 2.1. NMR 

**Figure 2:**
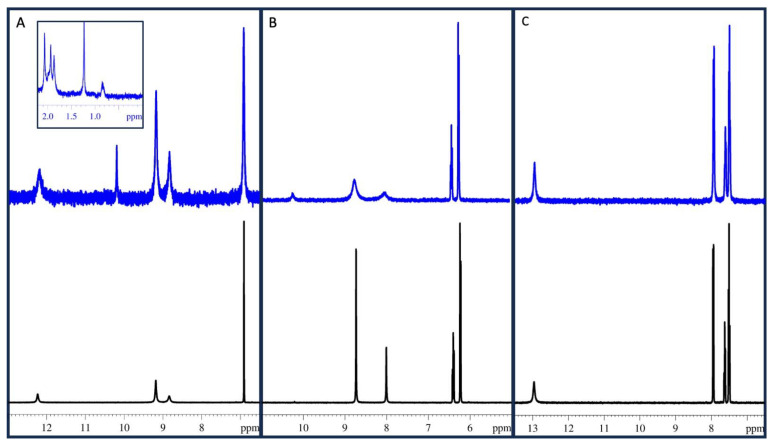
NMR spectra of unreacted (black) and reacted (blue) samples for gallic acid (**A**), pyrogallol (**B**), and benzoic acid (**C**). Unreacted samples were 1 mM. Reacted samples were a 1:1 molar ratio of phenol to Mn(IV) with a final concentration of 1 mM for each.

Figure 2 shows the NMR spectra of each compound before and after 2 h of reaction with Mn(IV). For gallic acid, the peaks representing the carboxylic acid (12.2 ppm), aromatic ^1^H (6.9 ppm), and hydroxyl groups (8.8 and 9.2 ppm) all diminished proportionally (Figure 2A). The NMR spectrum of the reacted sample had additional peaks not present in the unreacted sample, most notably at 10.2 ppm and multiple peaks in the 0–2.1 ppm range. Pyrogallol showed a change in the proportions of ^1^H after the reaction with Mn(IV) (Figure 2B). The proportion of the aromatic ^1^H (6–6.5 ppm) to hydroxyl ^1^H (8.0 and 8.7 ppm) changed from 1:1 to 1:1.4. Similar to the gallic acid reaction, the pyrogallol reaction sample also had an appearance of a new peak at 10.2 ppm; however, the pyrogallol reaction did not have any addition peaks in the 0–2.5 ppm range. Finally, benzoic acid did not show any significant difference between the reacted and unreacted samples.

### 2.2. Reverse-Phase HPLC

**Figure 3:**
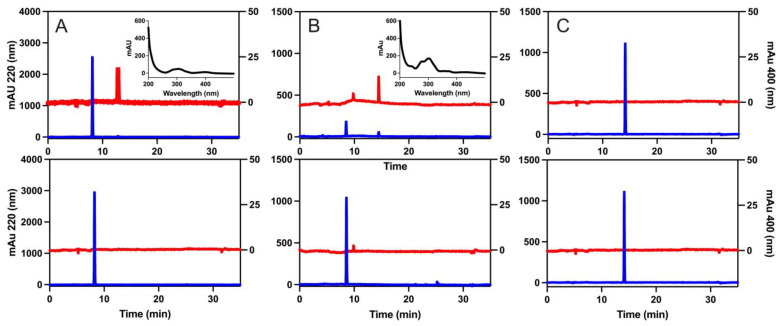
HPLC chromatograms for gallic acid (**A**), pyrogallol (**B**), and benzoic acid (**C**). Top panels represent the samples reacted with Mn(IV); bottom panels are unreacted samples. The detection wavelength 220 nm (left axis) is displayed in blue, and 400 nm (right axis) is displayed in red. Insert panels are full spectra of the peaks that appeared in the reacted samples. Unreacted samples were 1 mM. Reacted samples were in a 1:1 molar ratio with Mn(IV) with a final concentration of 1 mM for both.

Following the 2 h reaction, an average of 84.9% of the starting gallic acid remained (Table 1, Figure 3A). In addition to the gallic acid peak (8.2 min), there was an additional peak (12.8 min) in the reaction sample that absorbed at 400 nm. The full spectrum of this peak was captured (Figure 3A insert). Compared to gallic acid, more pyrogallol (8.5 min) was consumed in the reaction. Only an average of 21.2% of the pyrogallol remained after the 2 h reaction (Figure 3B, Table 1). The reaction sample had a new peak at 14.5 min that absorbed at 220 and 400 nm. The full spectrum of this peak was captured (Figure 3B insert). Finally, the benzoic acid (14.2 min) reaction had 97.7% of the starting compound remaining after 2 h (Figure 3C, Table 1).

### 2.3. CO_2_ Released

**Figure 4:**
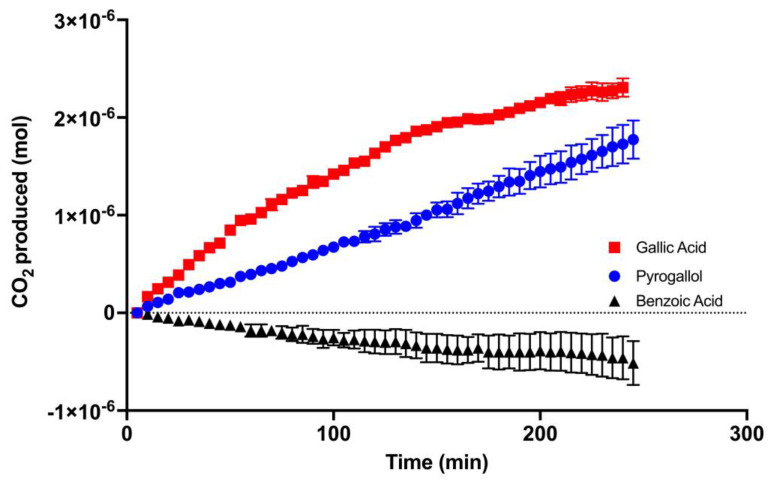
CO_2_ produced when Mn(IV) was reacted with gallic acid (red), pyrogallol (blue), and benzoic acid (black). Gallic acid and benzoic acid were reacted with Mn(IV) in a 1:1 molar ratio and a final concentration of 1 mM for both the phenol and Mn(IV). Pyrogallol was 1:1 with Mn(IV), and a final concentration of 5 mM for both. Final volume for all reactions was 50 mL. Values are means, error bars indicate the standard error of the mean, n = 3.

Figure 4 shows the amount of CO_2_ that was produced after each compound was reacted with Mn(IV). Gallic acid produced 2.3 × 10^−6^ mol of CO_2_ in the quart jar used for the experiment, while pyrogallol produced 1.8 × 10^−6^ mol of CO_2_. Benzoic acid did not produce CO_2_ during the time course of the experiment.

## 3. Discussion

The content of phenolic acids in soils and plant litter and the rate at which they degrade has been investigated [17,21], and many investigators have studied how phenolic acids interact with microorganisms [22,23]. The exact degradation products of phenolic acids have not been widely reported, likely due to experimental limitations owing to the complex nature of soils. In addition, more attention has been given to the concentration of phenolics in plants [17,24] or the biotic degradation of phenolic acids in soil environments [22,25], while few studies have investigated the abiotic process. In this study, we investigated the abiotic reactions of phenolic acids with Mn(IV). In our previous studies, the reaction with gallic acid and Mn(IV) approached completion by 3 h [20]. To ensure that we observed any potential intermediates, the samples were allowed a total reaction time of 2 h for the study described here. For the NMR analysis, this included the 30 min of shaking and 90 min of drying time on the speed vac. We also found in our earlier studies that the amount of CO_2_ produced increased as we increased the ratio of Mn(IV) to the phenolic acid. In the current study we maintained a 1:1 molar ratio because Mn, a paramagnetic metal, interferes with the NMR signal. The NMR and HPLC data (Figure 2 and Figure 3) indicate that in the presence of Mn(IV), gallic acid does not form pyrogallol via a decarboxylation reaction. The NMR spectrum of the gallic acid reaction suggests that the ring structure is likely breaking and producing small, aliphatic hydrocarbons. This is supported by the ratio of hydroxyl ^1^H to aromatic ^1^H pre- and post-reaction and the appearance of ^1^H in the alkane region. In contrast, pyrogallol had a change in the ratio of hydroxyl ^1^H to aromatic ^1^H from 1:1 to 1:1.4, suggesting that a quinone was being formed during the reaction. This conclusion is further supported by an appearance of a new peak at 14.5 min on HPLC that absorbed strongly in the 400 nm range. In our experiments, CO_2_ was produced when Mn(IV) was reacted with gallic acid or pyrogallol. To produce detectable levels of CO_2_ from pyrogallol, the concentration of the phenolic had to be increased fivefold over the typical concentrations used in these experiments. While more of the gallic acid remained (84.9%) after the reaction than pyrogallol (21.8%), gallic acid produced more CO_2_. This suggests different reaction rates and products for pyrogallol and gallic acid reactions with Mn(IV). Based on the ratio of aromatic to hydroxyl ^1^H following the reaction (Figure 2, Table 1), the product of the pyrogallol reaction is likely a quinone that was stable during the timeframe of our experiments. In contrast, gallic acid reacts less efficiently, but is fully oxidized to CO_2_. Benzoic acid did not show significant signs of a reaction in our experiments. Together, these data suggest that phenolic acids are able to be degraded abiotically by redox active metals in soil. Benzoic acid derivatives with phenolic hydroxyl groups are likely to produce at least some CO_2_.

Using gallic acid and pyrogallol as model compounds allows these data to be potentially extrapolated to higher-order phenolic compounds found in plants and soils. Gallotannins are polyphenols that are gallic acid derivates esterified to a sugar. In these structures, the hydroxyl groups are free, while the carboxylic acid is esterified to a sugar [26]. These compounds could undergo a similar reaction to the one we demonstrated here with pyrogallol. This suggests that simple benzoic acid derivatives with free carboxylic acid and hydroxyl groups are more likely to be oxidized and contribute less to SOM than larger phenols that are lacking a free carboxylic acid group. These large tannin-like structures are more likely to play a role in SOM for a longer time period than the simple phenolic acids.

## 4. Materials and Methods

### 4.1. Chemicals

Gallic acid was purchased from Acros Organics (Geel, Antwerp, Belgium), pyrogallol and benzoic acid were purchased from Alfa Aesar (Haverhill, MA, USA), Mn(IV) oxide (10 um) was purchased from Sigma Aldrich Co. (St. Louis, MO, USA), and methyl sulfide (DMSO)-d6 was purchased from Thermo-Fisher Scientific (Pittsburgh, PA, USA). Solutions of the phenols and Mn(IV) oxide were prepared in Milli-Q water by vortexing until dissolved. Gallic acid and benzoic acid required brief gentle heating. The pH was measured at the start of the reaction and after 2 h. The pH of the gallic acid reaction changed from 4.3 to 5.3, the pyrogallol reaction changed from 7.0 to 6.0, and the benzoic acid reaction was consistent at 3.9.

### 4.2. NMR Experiments

NMR characterization was performed on a Bruker Avance NEO 400 MHz spectrometer. ^1^H spectra were obtained at 400 MHz at room temperature. Reaction samples were prepared by mixing the phenolic acid with Mn(IV) oxide at a 1:1 molar ratio, with a final concentration of 1 mM for both the phenol and Mn(IV), and incubating at room temperature while shaking at 300 rpm for 30 min. Following the incubation, 800 μL of the reaction samples was dried on a speed vac and redissolved with 800 μL of DMSO-d6. The drying time on the speed vac was approximately 90 min, giving the reaction samples a total incubation time of 2 h. Phenolic acid standards were prepared as 1 mM solutions in Milli-Q water, then 800 μL of the phenol standards were dried in a speed vac and redissolved with 800 μL of DMSO-d6. Peaks on the NMR spectra were integrated using TopSpin software version 4.3.0.

### 4.3. Reverse-Phase HPLC Experiments

Phenolic acids were mixed with Mn(IV) oxide at a 1:1 molar ratio and allowed to incubate at room temperature while shaking at 300 rpm. A 500 mL subsample was removed after 2 h and filtered using a 0.22 μm cellulose acetate spin filter. All reactions were performed in triplicate. Phenolic acid concentrations before and after the reaction were quantified via reverse-phase HPLC using a diode array detector and a 3 um Hypersil GOLD C8 column (4.6 × 150 mm) at a flow rate of 0.5 mL min^−1^, using a gradient of 0.1% trifluoracetic acid (TFA) in water (A) and 0.1% TFA in acetonitrile (B) in a 35 min program as follows: 0–20 min, 2–98% (B), 20–25 min 98% (B). 25–30 min 98–2% (B), 30–35 min 2% (B). Detection wavelengths were 220 and 400 nm, and full absorbance spectra were captured of peaks as identified by the software. Peak identification and integration were performed using Agilent Chemstation software Rev A.09.03(1417).

### 4.4. CO_2_ Measurements

CO_2_ measurements were performed as previously described [20]. Briefly, reactions were placed in a 150 mL beaker inside a quart jar with a K30 CO_2_ sensor attached to an airtight lid. Samples were quickly added to the beaker, the lid to the quart jar was tightened, and the concentration of CO_2_ in the quart jar was logged via GasLab software version 2.3.1.4. For gallic acid and benzoic acid, reactions were prepared by mixing a 1:1 molar ratio of the phenol and Mn(IV), final concentration of 1 mM for both the phenol and Mn(IV), final volume 50 mL, and reactions were incubated at room temperature with constant stirring. The final pyrogallol concentration was 5 mM for both the phenol and Mn(IV). All other experimental conditions were identical to the other two compounds. All reactions were performed in triplicate. CO_2_ data were collected for 4 h, with measurements taken every 5 min.

## 5. Conclusions

In this study, we have provided a potential mechanism for how phenolic acids are abiotically degraded in soil via a reaction with Mn(IV). Our initial hypothesis, that Mn(IV) decarboxylates gallic acid to form CO_2_ and pyrogallol, was not supported. Instead, we demonstrated that hydroxybenzoic acid derivates undergo more complete oxidation, leading to the release of CO_2_. In this study, we also demonstrated that plant secondary metabolites lacking the carboxylic acid moiety are likely to be less oxidized in the short term and more likely to contribute to the formation of SOM.

## Figures and Tables

**Table 1:** NMR and HPLC results pre- and post-reaction.

	NMR Integration ^a^								HPLC
	Aromatic ^1^H		Hydroxyl ^1^H		Carboxyl ^1^H		Aromatic: Hydroxyl ^1^H		% Remaining after Reaction ^b^
Compound	Pre	Post	Pre	Post	Pre	Post	Pre	Post	
Gallic acid	0.11	0.011	0.15	0.015	0.050	0.005	1:1.5	1:1.5	84.9 (1.8)
Pyrogallol	1.02	0.055	1.00	0.040	N/A	N/A	1:1	1:1.4	21.8 (1.2)
Benzoic acid	0.23	0.11	N/A	N/A	0.036	0.018	N/A	N/A	97.7 (0.6)

^a^ Integration numbers represent the relative amount of each type of ^1^H. Values are summed for each type of ^1^H. ^b^ Amount of the starting compound that remained after the reaction with Mn(IV) expressed as a percentage. N/A = the compound does not contain that functionality. Values are means (standard error of the mean); n = 3.

## Data Availability

Data are contained within the article.
